# Nuclear Alarmin Cytokines in Inflammation

**DOI:** 10.1155/2020/7206451

**Published:** 2020-12-04

**Authors:** Lili Jiang, Yijia Shao, Yao Tian, Changsheng Ouyang, Xiaohua Wang

**Affiliations:** ^1^Department of Rheumatology and Clinical Immunology, Jiangxi Provincial People's Hospital Affiliated to Nanchang University, Nanchang, China; ^2^Department of Cardiology, Jiangxi Provincial People's Hospital Affiliated to Nanchang University, Nanchang, China; ^3^Geriatric Medical Center, Jiangxi Provincial People's Hospital Affiliated to Nanchang University, China

## Abstract

Pathogen-associated molecular patterns (PAMPs) are some nonspecific and highly conserved molecular structures of exogenous specific microbial pathogens, whose products can be recognized by pattern recognition receptor (PRR) on innate immune cells and induce an inflammatory response. Under physiological stress, activated or damaged cells might release some endogenous proteins that can also bind to PRR and cause a harmful aseptic inflammatory response. These endogenous proteins were named damage-associated molecular patterns (DAMPs) or alarmins. Indeed, alarmins can also play a beneficial role in the tissue repair in certain environments. Besides, some alarmin cytokines have been reported to have both nuclear and extracellular effects. This group of proteins includes high-mobility group box-1 protein (HMGB1), interleukin (IL)-33, IL-1*α*, IL-1F7b, and IL-16. In this article, we review the involvement of nuclear alarmins such as HMGB1, IL-33, and IL-1*α* under physiological state or stress state and suggest a novel activity of these molecules as central initiators in the development of sterile inflammation.

## 1. Introduction

The mechanism of the immune system sensing exogenous pathogens and internal tissue damage has attracted increasing attention. There are two main modes to activate the body's immune defense system when confronted with damage caused by various factors: one is ectogenic Pathogen-associated molecular patterns (PAMPs), and the other one is endogenic damage-associated molecular patterns (DAMPs) or alarmins. Pathogen-associated molecular patterns (PAMPs) are some nonspecific and highly conserved molecular structures that are necessary for the survival and pathogenicity of a class or a group of specific microbial pathogens [1]. Pattern recognition receptors (PRRs) are germline-encoded receptors that can recognize PAMP, thus triggers innate and adaptive immunity through activating a series of signaling pathways. One of the most important responses is to induce the synthesis of proinflammatory cytokines and the activation of inflammasomes downstream [2]. DAMPs or alarmins are endogenous proteins or peptides released by leukocytes and epithelial cells when stimulated by danger signals. They strengthen the innate and adaptive immunity by recruiting and activating the antigen-presenting cells (APCs) [3]. These DAMPs include high-mobility group box-1 (HMGB1), defensins, antimicrobial peptides, eosinophilic neurotoxins, heat shock proteins, and some cytokines like IL-1*α* and IL-33. It was thought that the biological effects of cytokines were only to transmit signals through specific receptors on the cell membrane, but increasing studies suggest that certain cytokines also play a role in the nucleus, such as IL-33, HMGB1, and IL-1*α* [4–6]. Here, we review the involvement of three representative nuclear alarmins, HMGB1, IL-33, and IL-1*α*, in the development of inflammation.

## 2. Members of Nuclear Alarmins Involved in Inflammation

### 2.1. HMGB1

**Figure 1:**
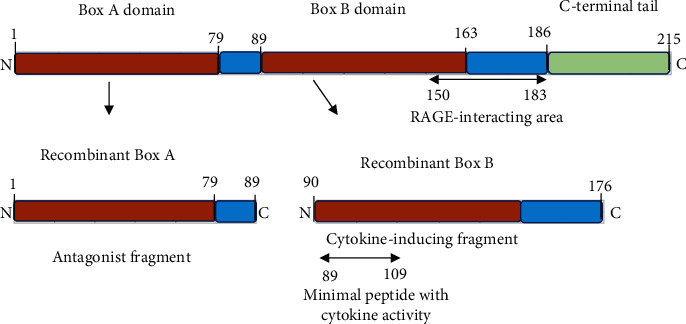
Molecular structure of HMGB1.

HMGB1 was named due to its low molecular weight and fast swimming in electrophoresis and was first recognized as an intranuclear protein [7]. It is present in almost all eukaryotic cells and is highly conserved between species [8]). Structurally, it is divided into three regions: Box A, Box B, and C-terminal domain. Both Box A and Box B are capable of binding to DNA; C-terminal is a residual terminal with a negative charge (Figure 1) [9]. Structure and function analysis showed that Box B had the biological activity of HMGB1, while Box A is an antagonist of HMGB1 and Box B, which can block the inflammatory effect of HMGB1 [10]. HMGB1 is a widely expressed nuclear protein and affects transcription regulation. It binds to the DNA grooves and loosens the DNA wrapped in the nucleosome, thus promoting chromatin remodeling [11]. HMGB1 can also bend the DNA significantly and promote the combination of DNA and relevant transcription factors, such as p53, NF-*κ*B, and steroid receptor [12, 13]. HMGB1-deficient mice die soon after birth suggesting the key role of HMGB1 in the nucleus in maintaining life [14]. HMGB1 stays very short at specific DNA binding sites and moves quickly in the nucleus. The stimulation of inflammation can lead to the acetylation of lysine residues in HMGB1 and prevent it from moving into the nucleus [15].

### 2.2. IL-33

**Figure 2:**
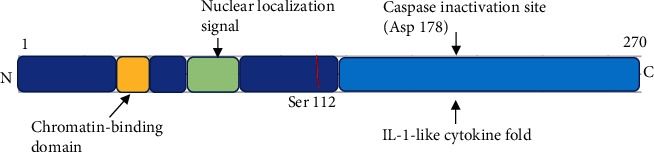
Molecular structure of IL-33.

Interleukin-33 (IL-33), also known as NF-HEV (nuclear factor from high endothelial venules), IL-1F11, is a new member of the IL-1 family originally reported by Schmitz et al. in 2005 [16]. It is widely expressed in the whole body, especially in the central nervous system and gastrointestinal [16]. It is composed of 270 amino acids, with an IL-1-like cytokine folding region at the C-terminal and a nuclear localization signal peptide and chromatin binding region at the N-terminal (Figure 2) [17]. IL-33 is synthesized at 30 KD in cellular and then cut into 18 KD by hydrolase as a mature form while secreted to extracellular [18]. Recent studies indicate that human IL-33 is processed at Asp178, not Asp112 as previously claimed [19, 20], and IL-33 is processed into bioactive forms and secreted to extracellular by neutrophil elastase and cathepsin G [21]. Recently, it has been reported that IL-33 is expressed in the nucleus, such as the human endothelial cells [22, 23]. The function of IL-33 in the nucleus is associated with the attachment to heterochromatin [24, 25].

IL-33 is derived from a wide range of tissues, but there are relatively few researches on which cell secreted IL-33 and its role in the disease. It has been reported that vascular endothelial cells (VECs) are the main source of IL-33. IL-33 are released from the nucleus when VECs are stimulated by inflammatory cytokines [26]. IL-33 is also expressed in the epithelial cells of the mucosa and the keratinocytes of skin [27–29], as well as some immune cells such as macrophages [30]. The secretory pathway of IL-33 is still unclear. It has been reported that it may be affected by the proteolytic enzyme, similar to that of IL-1*β* [31]. Researches also showed that cardiac fibroblasts stimulated by PMA and monocytes stimulated by LPS can secrete mature IL-33 [32, 33]. Recent studies suggested that the precursor IL-33 has biological activity, and its biological activity is reduced after proteolytic enzyme cleavage [19, 20, 30, 34].

### 2.3. IL-1*α*

**Figure 3:**
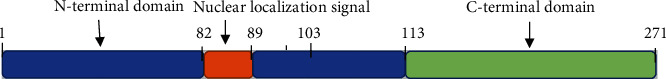
Molecular structure of IL-1*α*.

IL-1*α* is also an important member of the IL-1 family. IL-1*α* lacks secretory protein as a signal peptide, so it can only be transformed from its precursor molecule. When the cell is stimulated, proteases (calpain, Granzyme B, etc.) cut pro-IL-1*α* into the 17 kDa mature form of IL-1*α*, both of which have biological activities [35, 36]. Pro-IL-1*α* is a 31 KD protein, which can be expressed in most dormant nonhematopoietic cells of humans, such as the epithelial cells of the gastrointestinal tract, liver, kidney, and skin [37, 38]. It consists of the N-terminal domain (NTD), nuclear localization signal (NLS), and C-terminal domain (CTD) (Figure 3) [39]. NLS induced pro-IL-1*α* to migrate to the nucleus as an intranuclear transcription factor and participates in gene regulation [40, 41]. Mature IL-1*α* plays a biological role by binding to IL-1R [42].

## 3. Alarmin Receptors

Studies have shown that alarmins play a role in chemotaxis and activation of immune cells through G protein-coupled receptors (GPCRs) and non-G protein-coupled receptors (non-G PCR s) (Table 1).

### 3.1. Receptors for HMGB1

The Receptor for Advanced Glycation End Products (RAGE) is considered to be the receptor of HMGB1 [43]. RAGE is expressed on antigen-presenting cells (APC) [44–46], as well as endothelial cells and smooth muscle cells (SMCs) [47–51]. RAGE deficiency can significantly prolong the survival time of endotoxin mice. However, the deletion of RAGE does not completely prevent HMGB1 from stimulating macrophages to secrete inflammatory factors [52]. Other studies suggested that TLR2 and TLR4 are HMGB1's receptors as well [53]. However, there is no difference in the response of macrophages to HMGB1, whether the macrophages comes from TLR2-deficient mice or wild-type mice [54]. This suggests that TLR2, TLR4, and RAGE can bind to HMGB1, but RAGE may play a more important role for HMGB1.

Recent studies suggested that HMGB1 combined with other immune-stimulators, such as LPS, IL-1*β*, and DNA, can enhance its biological effect. This suggests that HMGB1 can simultaneously promote the activation of two receptors and produce biological effects. For example, HMGB1/DNA complex is easier to bind to RAGE than HMGB1, because the anchoring of DNA and TLR9 strengthens the combination of HMGB1 and RAGE [55, 56].

### 3.2. Receptors for IL-33

As the only specific receptor of ILl-33, ST2L is mainly expressed in Th2 lymphocytes [57], mast cells, and NKT, but not in Th1 lymphocytes [58]. IL-1 receptor accessory protein (IL-1RAcP) is essential for IL-33/ST2L to activate downstream signal pathways; IL-1RAcP-deficient mast cells cannot be stimulated to secrete IL-6 by IL-33 [59, 60]. IL-33 activates downstream signal pathways through ERK1/2, p38MAPK, and JNKs [16]; the TRAF6 pathway plays a key role in activating NF-*κ*B and inducing Th2 cytokines by IL-33 [61]. However, the relationship between ST2L and NF-*κ*B activation is controversial. It has been reported that the activation of ST2L has an anti-NF-*κ*B effect, as in cardiomyocytes; IL-33-activated NF-*κ*B inhibits angiotension II-induced NF-*κ*B activation, thus alleviating the cardiac hypertrophy [62–64]. Soluble ST2 (sST2) is the extracellular segment of ST2L, which acts as a decoy receptor and binds to IL-33 competitively, thus blocking the effect of IL-33 [65]. In animal experiments, injection of sST2 or ST2 blocking antibody can alleviate asthma mediated by IL-33 and block the proinflammatory effect of IL-33 on rheumatoid arthritis [66–68].

## 4. Alarmins in Inflammation

### 4.1. HMGB1 in Inflammation

HMGB1 shows a strong proinflammatory effect when released into the extracellular environment, mainly through the following two mechanisms. First, necrotic cells release HMGB1 and activate the immune system [69, 70]. Recent studies indicated that apoptotic cells can also release HMGB1, but the reactive oxygen species produced by the activation of intracellular hydrolase can inactivate HGMB1 and block its proinflammatory activity [71]. Second, monocytes or macrophages can secret HMGB1 when activated by LPS, proinflammatory factors, or NO [72]. In endotoxemia, HMGB1 is considered a lethal factor in the late stage of endotoxic shock [73–75]. Increasing inflammatory factors, such as LPS, TNF-*α*, and IL-1, induce macrophages or DCs secrete HMGB1, which further stimulates macrophages or DCs to secrete inflammatory factors, thus forming a vicious circle [76].

### 4.2. IL-33 in Inflammation

Recent studies suggested that IL-33 is involved in the occurrence and progress of various diseases, and its mechanism is complex. It can promote the pathophysiological progress of asthma [77, 78], rheumatoid arthritis [79], and systemic lupus erythematosus [80], while in atherosclerosis, allogeneic transplantation, endotoxic shock, and parasitic infection, it inhibits the occurrence and development of diseases [81].

The dual function of IL-33 is mainly due to the different types of immune responses on different cells. IL-33 induce Th2 cells [82], mast cells [83], and basophils to secrete large amounts of IL-4, IL-5, IL-13, IgE, and IgA [83], which induce the pathological changes related to Th2 immune response. In vivo administration of recombinant IL-33 can cause histological changes in the lung and gastrointestinal tract, such as increased mucus secretion, epithelial hyperplasia, and overgrowth, which were considered to be related to Th2 immune response induced by IL-33 [16]. Previous studies also reported that IL-33 can induce the tolerance of allografts, which may be related to the differentiation of Th2 cells, MDSCs, and Treg cells induced by IL-33 [84–86]. The specific role of IL-33 in the cell nucleus is still not very clear, but studies have suggested that it can regulate gene expression. First, IL-33 would be lost when stimulated by inflammation in the resting vascular endothelial cell (VEC) nucleus [22]; second, when binding to NF-*κ*B, IL-33 can block the related gene transcription induced by it [87]; and third, a short sequence of IL-33 precursor is involved in the formation of histone dimer, which is the components of higher-order chromatin structure [24].

### 4.3. IL-1*α* in Inflammation

IL-1*α* is an important alarmin that mediates aseptic inflammation. Studies have shown that the IL-1*α* expression can be upregulated in cells in the hypoxic environment, which activates aseptic inflammation. This is mainly due to the fact that hypoxia-inducible factor (HIF) induced by hypoxia can regulate the IL-1*α* transcription, thus affects the IL-1*α*-related inflammation by regulating the expression of IL-1*α* [88]. The expression and nuclear localization of IL-1*α* depend on the redox reaction. Overexpression of manganese superoxide dismutase leads to a corresponding increase of H2O2; meanwhile, a significant elevation of IL-1*α* is observed, in both mRNA and protein levels, as well as an increased localization of IL-1*α* in the nucleus [89].

## 5. Alarmins and Inflammatory Diseases

### 5.1. HMGB1 and Inflammatory Diseases

As a natural alarmin, HMGB1 is involved in the inflammatory response of acute local organ injury, as well as Th17-mediated autoimmune diseases, such as rheumatoid arthritis (RA), multiple sclerosis (MS), and its animal model-experimental autoimmune encephalomyelitis (EAE). HMGB1 is highly expressed in lesions of MS patients and EAE, and its three receptors RAGE, TLR2, and TLR4 are upregulated in macrophages or microglia. Besides, there is a positive feedback effect between HMGB1 and microglia, which promotes disease progression [90–92]. In the allograft rejection model, the expression of HMGB1 gradually increased over time. Notably, there was an ischemia-reperfusion injury in the process of obtaining the graft from the donor and during the surgery [93–96], which leads to the HMGB1 release from necrotic cells. These HMGB1 may be immediately involved in early and late graft rejection. The overexpression of HMGB1 is observed in colon cancer, breast cancer, and prostate cancer. With RAGE or HMGB1 blocked, tumor growth and metastasis are inhibited in animal models [97–99].

### 5.2. IL-33 and Inflammatory Diseases

In TNBS-induced enteritis, IL-33 upregulates CD103+IDO+ DCs through intestinal epithelial cells (IECs) and produces inhibitory Tregs to alleviate pathological changes mediated by Th1/Th17 [100]. IL-33 inhibits cardiac hypertrophy caused by AngII through activating NF-*κ*B, and as a decoy receptor of IL-33, the serum expression of sST2 increases in patients with myocardial hypertrophy and heart failure caused by it [62], and the expression was correlated with the grade of heart failure.

### 5.3. IL-1*α* and Inflammatory Diseases

IL-1*α* is an important dual inflammatory factor, mainly involved in a variety of autoimmune diseases, as well as in anti-infection, anti-tumor, and other processes [101]. By inducing the release of TNF-*α*, G-CSF, and other inflammatory factors and recruiting concentrated granulocytes [102, 103], IL-1*α* can promote the progress of acute lung injury [104, 105], DSS-induced intestinal inflammation, and psoriasis. In addition, IL-1*α* can also be used as a prognostic indicator for distant metastasis of head and neck squamous cell carcinoma and promote the growth of melanoma, pancreatic ductal adenocarcinoma, and other tumors [105, 106].

## 6. Conclusions

DAMP or alarmin is actively released by cells or directly released by necrotic tissues when the tissue is stimulated or damaged, then produce certain biological effects by binding to relative receptors. Alarmins may play different roles in different locations of cells or in the microenvironment of different diseases. Researchers hope to achieve the goal of curing diseases by regulating alarmins and their relative signal pathways. However, before achieving this goal, the mechanism of these cytokines still needs further research. Does DAMP affect each other? How is DAMP released from intracellular to extracellular? Is there any difference in the function between DAMP that is actively released or passively released? Are there any differences between DAMP that is released by apoptotic cells or necrotic cells? All in all, there is still a long way to go to clarify the biological effects and related mechanisms of DAMP.

## Figures and Tables

**Table 1:** Alarmins and relative receptors.

Alarmin	Relative receptors
HMGB1	RAGE, TLR2, TLR4
IL-33	ST2L
IL-1*α*	IL-1R
